# Some Considerations about the Use of Contact and Confocal Microscopy Methods in Surface Texture Measurement

**DOI:** 10.3390/ma11081484

**Published:** 2018-08-20

**Authors:** Jesús Caja García, Alfredo Sanz Lobera, Piera Maresca, Teresa Fernández Pareja, Chen Wang

**Affiliations:** 1 Department of Mechanical, Chemical and Industrial Design Engineering, ETS Ingeniería y Diseño Industrial, Universidad Politécnica de Madrid, Ronda de Valencia, 3, 28012 Madrid, Spain; piera.maresca@upm.es (P.M.); chen.wang@alumnos.upm.es (C.W.); 2 Department of Aerospace Materials and Production, ETSI Aeronáutica y del Espacio, Universidad Politécnica de Madrid, Plaza del Cardenal Cisneros, 3, 28040 Madrid, Spain; a.slobera@upm.es; 3 Department of Engineering Surveying and Cartography, ETSI en Topografía, Geodesia y Cartografía, Universidad Politécnica de Madrid, Campus Sur, Carretera de Valencia, Km 7, 28031 Madrid, Spain; teresa.fpareja@upm.es

**Keywords:** surface texture, contact measurement, optical measurement

## Abstract

Surface metrology employs various measurement techniques, among which there has been an increase of noteworthy research into non-contact optical and contact stylus methods. However, some deeper considerations about their differentiation and compatibility are still lacking and necessary. This work compares the measurement characteristics of the confocal microscope with the portable stylus profilometer instrumentation, from a metrological point of view (measurement precision and accuracy, and complexity of algorithms for data processing) and an operational view (measuring ranges, measurement speed, environmental and operational requirements, and cost). Mathematical models and algorithms for roughness parameters calculation and their associated uncertainties evaluation are developed and validated. The experimental results demonstrate that the stylus profilometer presents the most reliable measurement with the highest measurement speed and the least complex algorithms, while the image confocal method takes advantage of higher vertical and horizontal resolution when compared with the employed stylus profilometer.

## 1. Introduction

The evaluation of a surface texture involves the analysis of a large number of data using complex models [1]. For this purpose, metrology instruments must scan/measure the surface, obtaining a finite digital sample. Leach et al. [2] establish that the surfaces to be evaluated are defined by the measurement method used (measuring principle). The results will be different even when measuring the same surface with different instruments, since different physical properties are being measured. According to this idea, the instruments used in surface texture measurements can be classified into different groups, depending on the measurement technique and the physical property used to obtain the surface coordinates. When surface texture measurements are carried out on a mechanical manufacturing environment, the two main groups of instruments are based on tactile and optical methods.

In this context, tactile methods are mainly based on the use of stylus profilometers (SP), which are currently the most widely used instruments in the mechanical manufacturing industry [3]. In a common stylus profilometer, a probe, which is in contact with the surface, is physically moved over it, so that the vertical movement of the tip allows for characterization of the surface heights. This kind of instrument is preferably used in 2-D measurements based on profiles.

On the other hand, optical methods present a wide number of techniques for performing the measurement without surface contact using light instead of a physical probe to measure the surface [4]. The light reflected on the surface and its subsequent detection allows the evaluation of the surface texture. Conroy et al. [5] pointed out that the most widely used optical methods are interferometry and confocal microscopy (CM). The present work is focused on the second one, due to better adequacy when used in mechanical manufacturing environments.

The physical principle of confocal microscopy is based on eliminating the reflected light coming from the out-of-focus planes. The way to achieve this consists of illuminating a small area of the sample. The light beam from the focal plane is then taken so the beams from the lower and upper planes are removed by using a pin-hole. This confocal probe evaluates each point on the surface to be measured and obtains its height and associated light intensity. A system of lateral scanning allows having a line profile and areal measurement. The metrological characteristics of these devices are similar to those provided by the stylus profilometers and are gradually replacing them in specific metrological approaches [6].

On the other hand, different studies have evaluated and compared different techniques of surface metrology, emphasizing the characteristics of the equipment and the differences between the obtained surface parameters but not going into such detail in other non-metrological aspects related with the measurement.

Conroy et al. [5] measure a specimen consisting of an 80 μm pitch square wave Al-coated etched grating with a nominal step height of 187 nm and use stylus profilometers (SP), confocal microscopy (CM), and interferometric microscopy (IM) in their comparison. They do not provide experimental results of the evaluated surface texture parameters nor employed algorithms and conclude that the use of any technique requires an understanding of the properties of the sample, limitations of the technique used, and the analysis required before carrying out the surface measurement. Vorburger et al. [7] compare four techniques including stylus profiling (SP), phase-shifted microscopy (PSIM), white light interferometric microscopy (WLIM), and confocal microscopy (CM). They find discrepancies between WLIM and the other techniques, obtaining similar results among the other three. Poon et al. [8] compare three techniques—stylus profiler (SP), atomic force microscope (AFM), and non-contact optical profiler (NOP)—and conclude with a recommendation on the use of the analyzed techniques when a glass-ceramic substrate is measured. Nouira et al. [9] focused their work on the development of a high-precision profilometer with both optical (CM) and tactile capabilities and measures a VLSI Step-Height Standard (SHS 880-QC). Obtained results in their work show that the tactile measurements, which include stylus profilometers (SP) and atomic force microscopy (AFM), are more accurate than the optical measurements carried out by confocal microscopy (CM). The comparison of both tactile techniques reveals that the SP and the AFM measurements produce very similar results. Piska and Metelkova [10] analyze the relations between 2-D (profile) and 3-D (areal) surface parameters of the same measured surface, and they observed that both methods, SP and CM, give very comparable results only if the surface has a good reflection value. Nielsony et al. [11] analyze differences between a stylus profilometer (SP) and confocal microscopy (CM) in measuring a cladded surface, concluding that CM values of the roughness parameters are higher than SP values. The same result is obtained by Merola et al. [12] analyzing the tribological behavior of retrieved hip femoral head by using a stylus profilometer (SP) and confocal microscopy (CM). These variations in the results can affect the results of the surface topography [13,14,15], so the measurement principle should be close to the physical functional behavior of the surface [2].

Additional works have analyzed the behavior of optical instruments. Thompson et al. [16] make a quantitative comparison of areal topography measurements by using four optical techniques on a selective laser melting manufactured part. These techniques are confocal microscopy (CM), coherence scanning interferometry (CSI), focus variation microscopy (FVM), and X-ray computed tomography (XCT). The authors analyze the profile discrepancy between instrument pairs, obtaining high values (near 50%), due to the poorer capture of smaller scale peaks and pits of the FVM instrument. In the same line of work, Feidenhans’l et al. [17] compares optical methods for surface roughness measurement, employing different scatterometers and confocal microscopy (CM). The results are compatible between instruments, but it is necessary to include a Gaussian smoothing function to compensate for the differences.

All these results are interesting, although only partially covering the type of parts that the present work addresses, that is to say, those that are manufactured and used in mechanical manufacturing processes, such as machining processes. Moreover, algorithms or measuring procedures are weakly or not described in all that previous work. For these reasons, this work analyses and compares the application of both techniques, stylus profilometer (SP) and confocal microscopy (CM) measurements, in the evaluation of a series of surfaces, which include machined surfaces and two roughness standards (type C1 and C4 spacing standard), by establishing a comparison between them, and not only considering the results obtained (roughness parameters and their associated uncertainty), but also the procedure and the requirements and performances that these techniques need and offer. The work also considers other aspects related with the measurement, such as the set-up operations of pre-measurement samples, the operating time, operational considerations, data storage requirements, and the cost of instruments and maintenance.

## 2. Evaluation Procedure of Roughness Parameters

### 2.1. Parameters Calculation

In order to perform the proposed comparison, a specific procedure to evaluate the roughness parameters of a profile from its coordinates (x,z) has been developed. This way, when the results are compared, only the difference due to the type of instrument, and especially the data acquisition procedure, will be evaluated, which is not affected by systematic effects due to the calculation software that is different for each instrument.

The procedure of obtaining results is defined by the following steps:

1. Obtaining the extracted profile measured by different instruments. The file contains the sampled x coordinates and the digitized z coordinates $\left(x_{r},z_{r}\right)$.
2. Form removal. Due to the impossibility of placing the measured profile fully parallel to the measurement base, or the presence of geometric errors on the surface of the measurand, it is necessary to eliminate this form by fitting the data to a nominal shape (line, polynomial, and circle). Correction can be done in two ways: applying a tilt correction or by subtraction of the mean. This one will be used when the angle rotated by the surface is very small. The coordinates $\left(x_{rFr},z_{rFr}\right)$ are obtained. When the fitting to a regression line is employed, its mathematical representation is:
  (1) z=ax+b 
  The coefficients a and b can be calculated using the least squares method. The line that best fits the set of coordinates $\left(x_{i},z_{i}\right)$ is:
  (2) $$z_{i}-ax_{i}-b+e_{i}\approx 0\;$$
  where $e_{i}$ is the residual. It is possible to solve a linear system, obtaining the coefficients a and b, using:
  (3) $$P={\left(A\cdot A^{T}\right)}^{-1}\cdot A^{T}\cdot B\;\mathrm{where}\;A=\left[\begin{array}{cc} x_{1} & 1\\ \vdots & \vdots \\ x_{n} & 1 \end{array}\right]\;B=\left[\begin{array}{c} z_{1}\\ \vdots \\ z_{n} \end{array}\right]\;P=\left[\begin{array}{c} a\\ b \end{array}\right]\;$$
  Using the same procedure, it is possible to eliminate the form of surfaces that can be adjusted to quadratic polynomials.
3. $$\lambda_{s}$$ Filter. The profile is filtered using a low pass filter with a $\lambda_{s}$ cut-off. The primary profile is obtained with coordinates $\left(x_{rFrLs},z_{rFrLs}\right)$.
4. $$\lambda_{c}$$ Filter. The profile is now filtered using a high pass filter with a $\lambda_{c}$ cut-off according to the standard ISO 16610-21 [18]. This way, the waviness profile is eliminated, obtaining the roughness profile with coordinates $\left(x_{rFrLsLc},z_{rFrLsLc}\right)$.
5. Obtaining the roughness parameters, according to the standards ISO 4287 [19] and ISO 4288 [20]. In order to solve the calculation of the equations described in the aforementioned standards (roughness parameters *R_a_*, *R_q_*, *R_sk_*, and *R_ku_*), a method of integral analytical calculation, the trapezoidal method, has been used instead of the formula of discrete calculation (Equations (8)–(11)) to improve the accuracy of the results.
  (4) $$R_{p}=\underset{1\leq i\leq m}{\max}z_{pi}\;\;$$
  (5) $$R_{v}=\underset{1\leq i\leq m}{\max}z_{vi}\;\;$$
  (6) $$R_{z}=R_{p}+R_{v}\;\Rightarrow \mathrm{all}\;\mathrm{calculated}\;\mathrm{over}\;a\;\mathrm{sampling}\;\mathrm{length}\;$$
  (7) $$R_{t}=R_{p}+R_{v}\;\Rightarrow \mathrm{all}\;\mathrm{calculated}\;\mathrm{over}\;\mathrm{the}\;\mathrm{evaluation}\;\mathrm{length}\;$$
  (8) $$R_{a}=\frac{1}{l}\int_{0}^{l}\left|z\left(x\right)\right|dx\;\approx \;R_{a}=\frac{1}{n}\sum_{i=1}^{n}\left|z_{i}\right|\;$$
  (9) $$R_{q}=\sqrt{\frac{1}{l}\int_{0}^{l}z^{2}\left(x\right)dx}\;\approx \;R_{q}=\frac{1}{n}\sum_{i=1}^{n}z_{i}^{2}\;$$
  (10) $$R_{sk}=\frac{1}{R_{q}^{3}}\left[\frac{1}{l}\int_{0}^{l}z^{3}\left(x\right)dx\right]\;\approx \;R_{sk}=\frac{1}{R_{q}^{3}}\frac{1}{n}\sum_{i=1}^{n}z_{i}^{3}\;$$
  (11) $$R_{ku}=\frac{1}{R_{q}^{4}}\left[\frac{1}{l}\int_{0}^{l}z^{4}\left(x\right)dx\right]\;\approx \;R_{ku}=\frac{1}{R_{q}^{4}}\frac{1}{n}\sum_{i=1}^{n}z_{i}^{4}\;$$
  Parameters *R_t_*, *R_z_*, *R_p_*, and *R_v_* measure the amplitude of the profile (peak and valley distances), *R_a_* is used to calculate the average roughness, *R_q_* measures the variance of the amplitude distribution function (ADF) of the profile, *R_sk_* analyze the asymmetry of the ADF, and *R_ku_* evaluates the spikiness of the profile.
  Depending on the type of instrument evaluated, different coordinates (x,z) will be generated, so it will not be necessary to execute all steps.

### 2.2. Model for Calculating Uncertainties

Following the calculation model of roughness parameters described in the previous section, for the calculation of the uncertainty of these parameters, the Monte Carlo method is used. This numerical resolution method has a high application in metrological fields [21,22,23].

The documents Supplement 1 to the “Guide to the expression of uncertainty in measurement (GUM)”—Propagation of distributions using a Monte Carlo method [24] and Supplement 2 to the “Guide to the expression of uncertainty in measurement (GUM)”—Extension to any number of output quantities [25] have been used for the development of the calculation algorithms.

The calculation algorithms are provided in the following steps:

1. Definition of the output quantities. Parameters *R_t_*, *R_z_*, *R_p_*, *R_v_*, *R_a_*, *R_q_*, *R_sk_*, *R_ku_*, and *R_Sm_*.
2. Definition of input quantities. The sampled coordinates and the digitized coordinates, and scale division error of the instrument on the z-axis. In this work, only the sources that directly affect the coordinates of the captured profile, and therefore the variability of the measurements, have been taken into account.
  All of these magnitudes analyze the variations generated by the instrument in the measurements: noise in the readings, imperfections in the reference of the instrument, sampling and digitizing process, and rounding-off of the coordinates and software calculations, as well as the horizontal and vertical resolution of the instrument and the idealization of the Gaussian filter [26,27].
3. Assignment of the probability density functions (PDF) to the input variables. For the input variables defined above, it is established that variability due to the process of obtaining the sampled coordinates responds to a normal distribution with a mean of the raw coordinate value and a standard deviation equal to 2% of the sampling step [28,29].
  In order to analyze the variability of the z-coordinates, it was necessary to perform a repeatability study of the measurements [30]. That is to say, measuring the same profile a large number of times, by the same operator, with the same measurement procedure, same measuring system, and same operation conditions [31].
  After doing different experiments, it has been observed that it is practically impossible to obtain the same position of the z-coordinate to be analyzed. Therefore, an analysis of the repeatability of the parameters *P_p_* and *P_v_* is planned, where the parameters *P_p_* and *P_v_* are the maximum profile peak height of the primary profile and maximum profile valley depth of the primary profile, respectively. For this type C1 spacing standard, grooves having a sine wave profile will be measured and the standard deviations S of the previous parameters will be determined. This study assumes that this variability corresponds to a *t*-distribution (employed when a series of indications are evaluated). The standard uncertainty associated with this variability can be estimated as:
  (12) $$u\left(x\right)=\sqrt{\frac{\nu_{p}}{\nu_{p}-2}}\frac{S}{\sqrt{n}}\;$$
  where S is the maximum standard deviation of the parameters *P_p_* and *P_v_*, obtained in the experiment, $\nu_{p}$ is the degrees of freedom of the obtained parameters, and n represents the numbers of measurements made in the roughness measurement (typically one).
  Therefore, the digitized coordinates respond to a *t*-distribution of the mean value of the raw coordinate *z* and a standard uncertainty equal to the value calculated by the previous equation. The experimental values obtained are shown in Section 4.
  The scale division errors of the instrument on the z-axis, responds to a rectangular distribution of limits [−E,E], where E is the scale division on the z-axis.
4. Propagation. According to the recommendation of Supplement 1 to the GUM, in its Section 7.2.3, it is possible to use a small number of iterations (M) for complex models. Taking into account this recommendation, the standard deviation and the mean of the values obtained after performing the M iterations of the model, could be taken as u(y) and y respectively, and can be assigned to a Gaussian PDF $g_{Y}\left(\eta \right)=N\left(y,u^{2}\left(y\right)\right)$. In the simulations, 10,000 replications have been performed, requiring between 2 and 70 min of calculation on a computer with an i7-6700HQ-Intel(R) Core(TM) and 16 GB memory.
5. Results. The standard deviation of the resulting values obtained in the simulations, as well as their mean, is calculated. To determine the amplitude of the coverage interval, the minimum interval method is used [32,33]. Using the suggestions of Supplement 2 of the GUM, it is possible to determine the covariance matrix C of the calculated parameters (all parameters will be correlated to a certain degree, due to the presence of common input variables in all of them). From the covariance matrix it is possible to calculate the matrix of correlation coefficients r.

### 2.3. Algorithm Validation

In order to determine the validity and accuracy of the developed algorithms, both reference and synthetic data sets [34] have been used. These data are the coordinates (x,z) of a series of points linked to a reference profile. The reference data have been obtained from the Internet-based Surface Metrology Algorithm Testing System of the National Institute of Standard and Technology (NIST) [29]. These simulation-generated profiles and F1-type software standards have been generated in accordance with the specifications of ISO 5436-2 [35].

**Figure 1:**
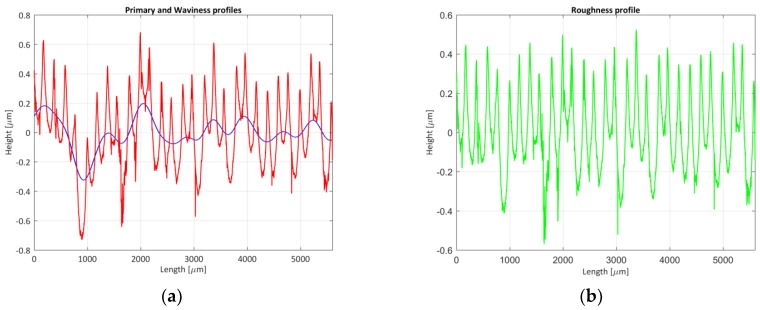
Mill F1-type (**a**) primary and waviness profile; (**b**) roughness profile.

One of the verifications performed in this work is shown in Table 1. The F1-type standard Mill.sdf file [29] shown in Figure 1 has been used and its roughness parameters have been evaluated using a cut-off ($\lambda_{c}$) of 0.8 μm and a Gaussian filter according to ISO 16610-21 [18] (steps 4 and 5).

In order to determine how good the developed algorithms are, the calculated results with the reference data are compared with NIST data. The difference, in absolute value, between the parameters obtained by the proposed algorithms and the reference parameters is used.

(13) $$Q_{1}=\left|R_{}^{calculated}-R_{}^{reference}\right|\;$$

In view of the results, it can be observed that the maximum percentage difference was 0.0061%, which is the reason why we claim the developed algorithms behave in a totally satisfactory manner. In addition, the algorithms of form elimination and low pass filtering for $\lambda_{s}$ (steps 2 and 3) using the software provided by the Physikalisch-TechnischeBundesanstalt (PTB) “Software to Analyse Roughness of Profiles-Version 2.09” [36] have been validated, since they provide similar results to those shown. A Type D roughness standard has been measured, and the data of the extracted profile has been introduced in the previous software, obtaining the following results (Table 2).

## 3. Methodology of Experimental Study on Roughness

**Figure 2:**
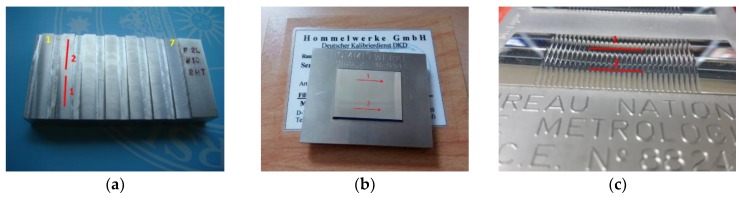
(**a**) Measurands 1 to 7; (**b**) Measurand 8; (**c**) Measurand 9.

To perform the comparison of measurement characteristics between the image confocal microscope and some portable stylus measurement instrumentations, three specimens are measured. The first one is a milled part with the same tool and with different feed per tooth (measurand 1 to 7); The second one is a Type C1 spacing standard with grooves having a sine wave profile (measurand 8); The third one is a Type C4 spacing standard with grooves having an accurate profile (measurand 9). Figure 2 shows the three specimens, as well as their measurement areas.

**Figure 3:**
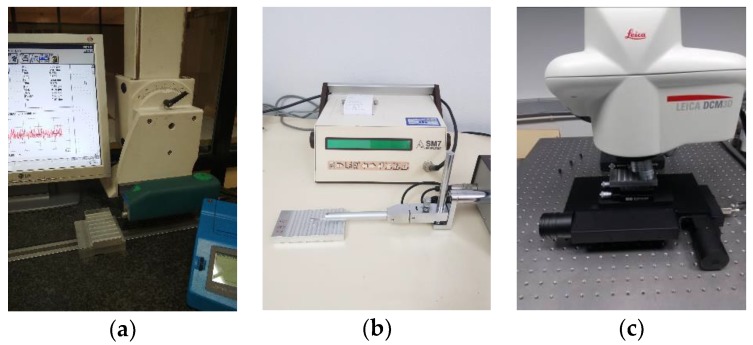
Experimental setup. (**a**) *SP-1*; (**b**) *SP-2*; (**c**) *CM-3*.

The technical features of the three instruments used in the comparison are detailed below:

- Stylus profilometer 1 (*SP-1*): portable stylus profilometer with a mechanical probe, brand HOMMELWERKE, model TESTER T1000 WAVE with probe TKL 300L, stylus tip of 5 µm/90°. Measuring range: ±80 µm/±320 µm. Vertical resolution (Z): 0.01 µm/0.04 µm. Transverse length: 0.05–20 mm. X sampling: 0.583 µm (Figure 3a).
- Stylus profilometer 2 (*SP-2*): portable stylus profilometer with a mechanical probe, brand SM, model PROFILTEST SM7 with stylus tip of 5 µm. Measuring range: 320 µm. Vertical resolution (Z): 0.01 µm. X sampling: 2.5 µm (Figure 3b).
- Confocal microscope (*CM-3*) confocal microscope, brand Leica (Wetzlar, Germany), model DCM-3D. It uses episcopic illumination, with light source LED type of wavelength 460 nm. Image Acquisition Sensor: monochrome CCD for confocal applications. The equipment has five lenses, with amplification between 5× and 150×. A 50× lens was used in the measurements to provide agreement between amplification and data acquisition speed. This lens has the following characteristics: Measuring field: 254.64 × 190.90 µm. Pixel size: 0.332 µm and equal to the lateral resolution (XY). Vertical resolution (Z): <3 nm. Total measuring field of instrument: 114 × 75 × 40 mm (Figure 3c).

## 4. Analysis of Experimental Results

The main aspects, as defined in Section 1, are analyzed below and will serve as a comparison of the evaluated methods, tactile and optical.

First, the repeatability study of the measurements made with the three instruments used in the comparison is developed. Measurand 8 is measured 25 times. Table 3 establishes the values obtained in this study. The standard deviations are similar for the three instruments, although the confocal microscope (*CM-3*) has the higher vertical resolution.

### 4.1. Tactile Method

**Figure 4:**
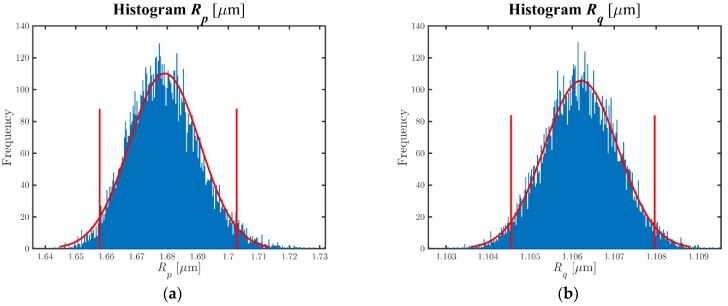
(**a**) *R_p_* histogram; (**b**) *R_q_* histogram.

- Measuring ranges: In height (z-axis), the smallest measuring field allowed by each instrument has been selected. In length (x-axis), a profile of length of 5.6 mm (7× $\lambda_{c}$) has been evaluated.
- Operating time: The effective measurement time, which included the movement of the probe on the surface, the calculation of parameters by the equipment, and the saving of the data files, has been less than 2 min. The preparation time of the sample, which includes the positioning and inclination adjustment by rotation of the *SP-1* probing system (Figure 3a), lasted 10 min at most.
- Environmental considerations: Regarding the *SP-1* and *SP-2* environmental requirements, the measurements were carried out at a controlled temperature of 20 ± 1 °C
- Measurand preparation: It included the cleaning of the measurand by a mixture of alcohol and ether, as well as the correct placement of the measurand on the basis of measurement (indicated in the previous point). Prior to measurement, the part had been thermally stabilized for at least 3 h.
- Numeric values of the parameters: Table 4 shows, as an example, the results of measurand 8 when it was measured with the *SP-1* equipment for a simulation performed for M = 10^4^. Figure 4a,b show, by way of example, the histograms of parameters *R_p_* and *R_q_*. It was verified that these parameters can be reasonably approximated to a normal distribution. Analogous behavior was obtained for the other parameters. Table 5 shows the correlation coefficients matrix of the roughness parameters, verifying the presence of correlation between them. Some of them show high correlation coefficient values (>0.5), due to the fact that all the parameters employ the same input quantities, that is to say, raw coordinates $\left(x_{r},z_{r}\right)$. Table 6 (columns 2 and 4) and Table 7 (columns 2 and 4) show some of the values obtained in the measurements of the different measurands after applying the algorithm used for the evaluation of roughness parameters. As it can be observed, there were discrepancies between the values of the equipment, higher for the amplitude parameters (peak and valley), presenting differences between 11% and 17% of the value analyzed and minimal when evaluating the amplitude parameter (average of ordinates), with differences in percentage between 0.2% and 4%. The difference in the spacing parameter was due to the different x sampling value of each piece of equipment. When comparing measurands, better amplitude parameter results (average of ordinates) were obtained when a standard was evaluated (measurand 8). When the piece was measured, differences up to 12% were obtained due to the impossibility of measuring the same profile with different equipment, and the presence of irregularities in the surface of the piece. The uncertainty of the parameters was similar when a standard or a piece was measured. The amplitude parameters (peak and valley) presented values of uncertainty of hundredths of a micrometer and were at least one or two orders of magnitude higher than the uncertainty of the amplitude parameters (average of ordinates). This was due to the process of obtaining these parameters.
- Data storage requirements: The data file, ASC/.txt extension, presented 9598/2241 rows of values, providing coordinates $\left(x_{r},z_{r}\right)$/step and $z_{r}$/z coordinates, with a file size of 260/25 kB (*SP-1*/*SP-2*).
- Cost of instrumentation/maintenance: The acquisition cost of the equipment was 12,000/6000 €. The maintenance was practically non-existent (lubrication of the vertical slide guides of the probing head for the *SP-1* equipment).

### 4.2. Confocal Microscope

**Figure 5:**
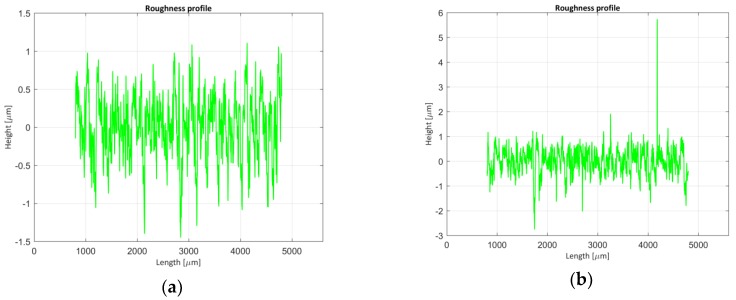
Roughness evaluated profile in measurand 2 (**a**) *SP-1*; (**b**) *CM-3*.

**Figure 6:**
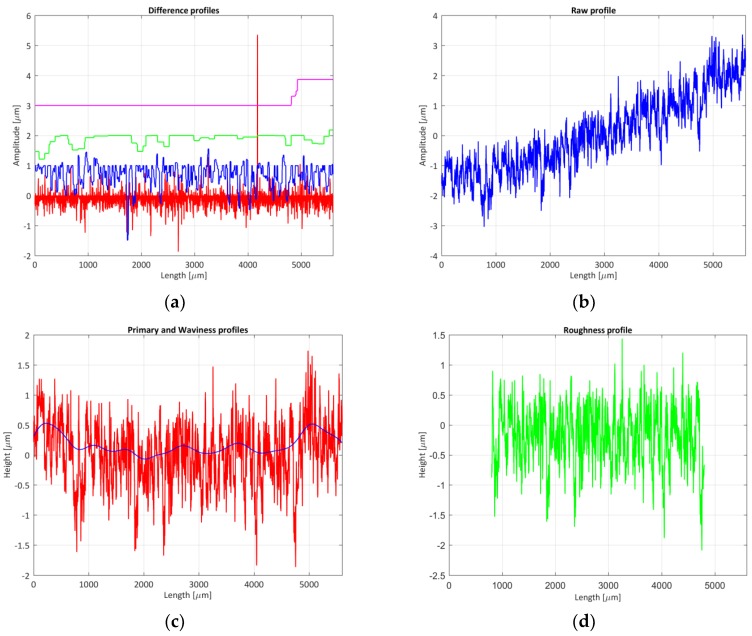
Roughness evaluated profile with a confocal microscope (outliers eliminated): (**a**) morphological filter application; (**b**) raw profile obtained after filtration; (**c**) waviness profile; (**d**) roughness profile.

- Measuring ranges: The evaluation field of 100 µm was selected. In length (x-axis), a profile of length 5.6 mm (7× $\lambda_{c}$) was evaluated. Because the field of measurement of the objective was less than the total length of scanning, 25 measurement fields had to be evaluated and the overlapping images (stitch) had to be implemented. The option of “profile measuring” was used in the instrument, obtaining only coordinates $\left(x_{r},z_{r}\right)$, keeping the coordinate $y_{r}$ constant.
- Operating time: The effective measurement time, which included the measurement of the 25 measurement fields on the surface, the calculation of parameters by the equipment and the saving of the data files, was about 10 to 15 min. Sample preparation time, which included the placement and inclination adjustment by a tilt table (Figure 3b), lasted between 15 and 20 min.
- Operational considerations: There was a significant probability of obtaining outliers or points not measured in the sample, so it was necessary to repeat the measurement a high number of times. Unmeasured points appeared when the Charge-Coupled Device (CCD) sensor of the confocal microscope did not receive enough light intensity to detect a peak position. This fact could be caused by local slope effects, which meant that the reflected light was not picked up by the target, or that the light intensity selected for the measurement was not adequate (low). On the contrary, if the luminous intensity of the equipment was very high, the CCD sensor could became saturated, tampering the peak position, and obtaining incorrect z-coordinate values. These effects could produce sharp peaks and valleys which were not real, as Figure 5b shows. Also, due to the geometry of the roughness profile, the illumination beam could only solve slopes with a maximum angle of 90 degrees.
  Very fine adjustment of the light intensity used in the measurement, and even the creation of different levels of illumination according to the depth of the sample, were therefore necessary. [37]. Likewise, it was be necessary to correct the unmeasured points and outliers by using interpolation and filtering techniques, respectively.
- Environmental considerations: The equipment was installed in the *Laboratorio de Investigación de Materiales de Interés Tecnológico* (LIMIT) laboratory of *ETS de Ingeniería y Diseño Industrial* (ETSIDI). The measurements were carried out at a controlled temperature of 20 ± 1 °C.
- Measurand preparation: The same as in the tactile instrument.
- Numeric values of the parameters: Table 6 (columns 6 and 7) and Table 7 (columns 6 to 9) show the roughness parameters obtained when evaluating measurands 8 and 2 respectively. If the results are compared when measuring the same sample with the stylus profilometers (Section 4.1), there are discrepancies between both methods, due to the causes mentioned above (outliers and not-measured points). When the standard was measured, differences of values of the parameters of between 2% and 15% were obtained. These differences could reach 300% when the piece was measured. In both cases, the smallest differences between the roughness parameter were obtained when the amplitude parameter (average of ordinates) was compared. As other authors have verified, most of the roughness parameters measured with the confocal microscope show higher values than those obtained with the profilometers. The uncertainty of the parameters showed the same order the magnitude as those obtained with the tactile instruments. To solve the problem of discrepancies, a morphological profile filter was employed: scale space techniques according to ISO 16610-49:2015 [38] so that it was possible to smooth the profile using different circular disks [39] and the outliers would be eliminated. Figure 6a,b show the results obtained when applying this technique. Also, it was decided to use a robust Gaussian regression filter [39,40] in order to improve the results, defined in standard ISO 16610-31:2016 [41]. This way, the waviness profile would not be affected by possible outliers that were not eliminated in the previous step. Figure 6c shows the primary profile and the waviness profile, and Figure 6d shows the roughness profile. By applying these modifications, it was possible to observe how the results obtained improve considerably when implementing the previous techniques, Table 6 (column 9), obtaining a maximum difference, when the *R_sk_* parameters evaluated at 50% of its value (without the filtering technique the difference was 240%). As an average value, the difference between parameters was 14% and 105% without the filtering technique.
- Data storage requirements: The data file had 16,885 points with coordinates $\left(x_{r},\;z_{r}\right)$ and a size of 310 kB.
- Cost of instrumentation/maintenance: The acquisition cost of the equipment was approximately 120,000 €. Maintenance was practically non-existent (replacement of LED light sources after about 10,000 h of operation).

**Figure 7:**
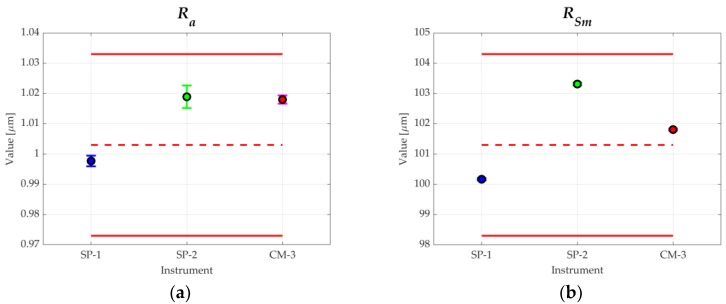
Comparison of results for: (**a**) *R_a_*; (**b**) *R_Sm_*.

Finally, the compatibility between the results of the roughness parameters obtained by the different measurement methods and instruments and the values provided by the calibration certificate of the type C1 spacing standard (measurand 2) were analyzed. The calibrated parameters of the standard give the next values: Parameter *R_a_* = 1.003 μm with an expanded uncertainty (*k* = 2) of 3% of the value of the parameter; Parameter *R_Sm_* = 101.3 μm with an expanded uncertainty (*k* = 2) of 3 μm. When comparing the previous values with the uncertainty of the obtained measurements, it could be affirmed that the results were coherent with the certified values (the measured values were contained in the interval characterized by the certified value and its expanded uncertainty). Figure 7a,b shows this consideration. It was verified that the values of the analyzed roughness parameters, obtained with the confocal microscope, always exceeded the certified values.

It should be noted that the present work has used parts that could be measured by any of the two considered techniques, optical or tactile. Therefore, the size and location of the measured parts had not been taken into account. Both size and location were factors of great relevance, which in many situations determine the type of measurement to be performed. In this sense, contact measurement techniques were usually advantageous compared to optical techniques due to the wide variety of existing profilometers, their portability, and the possibility of working in less restrictive environmental conditions.

## 5. Conclusions

This work has developed a comparative study between two kinds of measurement techniques that can be used in surface metrology, namely, two portable stylus profilometers (tactile) and a confocal microscope (optical). Measurement results, needed requirements, and available performances of both techniques have been analyzed. Both the metrological characteristics (measurement precision and accuracy, complexity of algorithms for data processing, etc.) and the operational characteristics (measuring ranges, measurement speed, environmental and operational requirements, costs, etc.) were studied.

The algorithms and procedures for calculating the uncertainties associated with the roughness parameters have been developed by employing the Monte Carlo method. The correlations between parameters have been found.

Measurements of several measurands obtained with the portable stylus profilometer have been shown to be the most reliable method when compared with the certified values of the measured standard, in line with Leach conclusions [2]. The procedure required little preparation and measurement time, and the measurement algorithms were simple. As a disadvantage, the low vertical resolution, i.e., the z-value, of the two instruments employed when compared to optical methods, could be neglected. With respect to the low x sampling value of the portable stylus profilometer employed, if compared with the confocal microscope, it could also be neglected. This conclusion, for other types of stylus profilometers with better metrological characteristics *r* (higher lateral resolution) that were not analyzed in this paper, may not be true.

The use of a confocal microscope allows for improving the previous parameters (lower uncertainty) at the expense of greater measurement and preparation times, as well as a very high cost of equipment. Besides, it forced the development of more complex algorithms due to the presence of outliers and points not measured on the sample in order to obtain reliable results. From the experimental study, it had been observed that when the confocal microscope was employed, it was possible to reduce nearly 10 times the difference between parameters (compared with results obtained with a stylus profilometer) when the profile was filtered employing a morphological profile filter and a robust Gaussian regression filter.

Further studies would be suitable in order to include more terms that contribute to the uncertainty of the roughness parameters (standard contribution and sensor interaction (light or tip) among others).

## Figures and Tables

**Table 1:** Reference values vs calculated values (NIST Mill measured profile).

Parameter	Reference Value	Calculated Value	*Q*_1_ (×10^−6^)	Percentage Difference (%)
*R_t_* [μm]	1.09408	1.09408	1.5	0.0001
*R_z_* [μm]	0.89833	0.89833	1.9	0.0002
*R_p_* [μm]	0.46672	0.46672	4.0	0.0008
*R_v_* [μm]	0.43161	0.43161	2.0	0.0005
*R_a_* [μm]	0.16764	0.16765	10.3	0.0061
*R_q_* [μm]	0.20479	0.20479	2.2	0.0011
*R_sk_* [-]	0.1388	0.1388	3.5	0.0025
*R_ku_* [-]	2.37947	2.37947	6.6	0.0027
*R_Sm_* [μm]	255.82	255.83	6542.4	0.0025

**Table 2:** Reference values vs. calculated values (PTB’s “Software to Analyse Roughness of Profiles”).

Parameter	PTB Reference Value	Calculated Value	*Q*_1_ (×10^−3^)	Percentage Difference (%)
*R_t_* [μm]	2.0468	2.0489	2.1	0.1026
*R_z_* [μm]	2.0037	2.0051	1.4	0.0699
*R_p_* [μm]	1.0109	1.0116	0.7	0.0692
*R_v_* [μm]	0.9928	0.9935	0.7	0.0705
*R_a_* [μm]	0.8943	0.8947	0.4	0.0447
*R_q_* [μm]	0.9068	0.9071	0.3	0.0331
*R_sk_* [-]	0.0107	0.0110	0.3	2.8037
*R_ku_* [-]	1.0516	1.0511	0.5	−0.0475
*R_Sm_* [μm]	80.96	80.89	70	−0.0865

**Table 3:** Results of the repeatability study.

Instrument	Standard Deviation *R_p_* (μm)	Standard Deviation *R_v_* (μm)
*SP-1*	0.0685	0.0584
*SP-2*	0.0539	0.0747
*CM-3*	0.0545	0.0707

**Table 4:** Results of the simulations of the eight measurand with the *SP-1* equipment.

Roughness Parameter	Parameter Estimation *y*	Standard Uncertainty *u*(*y*)	Shortest 95% Coverage Interval	
			Lower Limit	Upper Limit
*R_t_* [μm]	3.433	0.032	3.375	3.496
*R_z_* [μm]	3.367	0.016	3.336	3.399
*R_p_* [μm]	1.679	0.012	1.658	1.703
*R_v_* [μm]	1.688	0.012	1.667	1.711
*R_a_* [μm]	0.9977	0.0009	0.9960	0.9993
*R_q_* [μm]	1.1062	0.0009	1.1045	1.1080
*R_sk_* [-]	0.0019	0.0016	−0.0012	0.0052
*R_ku_* [-]	1.4860	0.0015	1.4829	1.4888
*R_Sm_* [μm]	100.167	0.018	100.142	100.206

**Table 5:** Correlation coefficient matrix of the roughness parameters.

Roughness Parameter	*R_t_*	*R_z_*	*R_p_*	*R_v_*	*R_a_*	*R_q_*	*R_sk_*	*R_ku_*	*R_Sm_*
*R_t_*	1.00	0.72	0.51	0.51	0.08	0.11	−0.01	0.14	0.00
*R_z_*	0.72	1.00	0.71	0.71	0.12	0.17	−0.01	0.22	−0.01
*R_p_*	0.51	0.71	1.00	0.00	0.09	0.12	0.12	0.14	−0.02
*R_v_*	0.51	0.71	0.00	1.00	0.08	0.12	−0.14	0.17	0.00
*R_a_*	0.08	0.12	0.09	0.08	1.00	0.91	0.00	−0.29	−0.04
*R_q_*	0.11	0.17	0.12	0.12	0.91	1.00	0.01	0.01	−0.03
*R_sk_*	−0.01	−0.01	0.12	−0.14	0.00	0.01	1.00	−0.01	−0.01
*R_ku_*	0.14	0.22	0.14	0.17	−0.29	0.01	−0.01	1.00	0.01
*R_Sm_*	0.00	−0.01	−0.02	0.00	−0.04	−0.03	−0.01	0.01	1.00

**Table 6:** Roughness values obtained with the different instruments and methods (measurand 8).

Instrument	*SP-1*		*SP-2*		*CM-3*	
	Parameter Estimation	Standard Uncertainty	Parameter Estimation	Standard Uncertainty	Parameter Estimation	Standard Uncertainty
*R_t_* [μm]	3.433	0.032	4.015	0.074	3.831	0.034
*R_z_* [μm]	3.367	0.016	3.781	0.036	3.686	0.017
*R_p_* [μm]	1.679	0.012	1.866	0.025	1.947	0.012
*R_v_* [μm]	1.688	0.012	1.915	0.026	1.739	0.011
*R_a_* [μm]	0.9977	0.0009	1.0189	0.0019	1.0180	0.0007
*R_q_* [μm]	1.1062	0.0009	1.1370	0.0020	1.1309	0.0007
*R_sk_* [-]	0.0019	0.0016	−0.0455	0.0039	0.0406	0.0013
*R_ku_* [-]	1.4860	0.0015	1.4828	0.0044	1.5104	0.0012
*R_Sm_* [μm]	100.167	0.018	103.3108	0.027	101.808	0.017

**Table 7:** Roughness values obtained with the different instruments and methods (measurand 2).

Instrument	*SP-1*		*SP-2*		*CM-3 (With Outliers)*		*CM-3 (Outliers Removed)*	
	Parameter Estimation	Standard Uncertainty	Parameter Estimation	Standard Uncertainty	Parameter Estimation	Standard Uncertainty	Parameter Estimation	Standard Uncertainty
*R_t_* [μm]	2.613	0.046	2.939	0.088	8.484	0.044	3.442	0.090
*R_z_* [μm]	2.243	0.022	2.350	0.038	4.061	0.019	2.632	0.029
*R_p_* [μm]	1.010	0.016	1.064	0.027	2.250	0.014	1.295	0.018
*R_v_* [μm]	1.233	0.016	1.286	0.027	1.811	0.014	1.337	0.023
*R_a_* [μm]	0.3300	0.0008	0.3697	0.0018	0.39844	0.00063	0.3732	0.0018
*R_q_* [μm]	0.4186	0.0008	0.4661	0.0019	0.53093	0.00065	0.4660	0.0011
*R_sk_* [-]	−0.2420	0.0084	−0.4338	0.0168	0.338	0.012	−0.375	0.010
*R_ku_* [-]	3.0868	0.0191	3.0862	0.0415	12.79	0.13	3.107	0.027
